# Metal-Free Biomass-Derived Environmentally Persistent Free Radicals (Bio-EPFRs) from Lignin Pyrolysis

**DOI:** 10.1021/acsomega.2c03381

**Published:** 2022-08-16

**Authors:** Lavrent Khachatryan, Mohamad Barekati-Goudarzi, Rubik Asatryan, Andrew Ozarowski, Dorin Boldor, Slawomir M. Lomnicki, Stephania A. Cormier

**Affiliations:** † Department of Chemistry, Louisiana State University, Baton Rouge, Louisiana 70803, United States; ‡ Department of Chemical and Biological Engineering, University at Buffalo, The State University of New York, Buffalo, New York 14260, United States; § National High Magnetic Field Laboratory, 1800 East Paul Dirac Drive, Florida, Tallahassee 32310, United States; ∥ Department of Biological and Agricultural Engineering, LSU AgCenter and LSU A&M College, Baton Rouge, Louisiana 70803, United States; ⊥ Department of Environmental Sciences, Louisiana State University, Baton Rouge, Louisiana 70803, United States; # Department of Biological Sciences, LSU Superfund Research Program and Pennington Biomedical Research Center, Baton Rouge, Louisiana 70808, United States

## Abstract

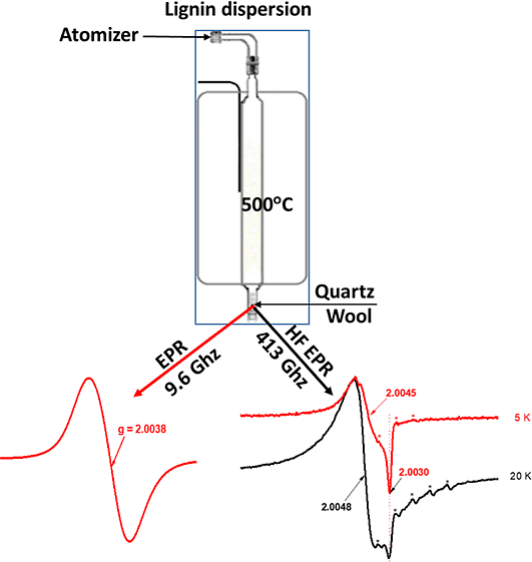

To assess contribution of the radicals formed from biomass burning, our recent findings toward the formation of resonantly stabilized persistent radicals from hydrolytic lignin pyrolysis in a metal-free environment are presented in detail. Such radicals have particularly been identified during fast pyrolysis of lignin dispersed into the gas phase in a flow reactor. The trapped radicals were analyzed by X-band electron paramagnetic resonance (EPR) and high-frequency (HF) EPR spectroscopy. To conceptualize available data, the metal-free biogenic bulky stable radicals with extended conjugated backbones are suggested to categorize as a new type of metal-free environmentally persistent free radicals (EPFRs) (bio-EPFRs). They can be originated not only from lignin/biomass pyrolysis but also during various thermal processes in combustion reactors and media, including tobacco smoke, anthropogenic sources and wildfires (forest/bushfires), and so on. The persistency of bio-EPFRs from lignin gas-phase pyrolysis was outlined with the evaluated lifetime of two groups of radicals being 33 and 143 h, respectively. The experimental results from pyrolysis of coniferyl alcohol as a model compound of lignin in the same fast flow reactor, along with our detailed potential energy surface analyses using high-level DFT and ab initio methods toward decomposition of a few other model compounds reported earlier, provide a mechanistic view on the formation of C- and O-centered radicals during lignin *gas-phase* pyrolysis. The preliminary measurements using HF-EPR spectroscopy also support the existence of O-centered radicals in the radical mixtures from pyrolysis of lignin possessing a high *g* value (2.0048).

## 1 Introduction

**Figure 1:**
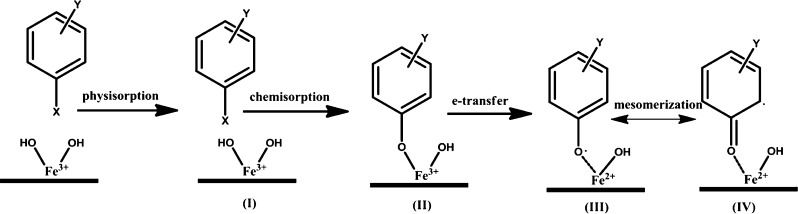
General mechanism (LSU model) of the formation of EPFRs on a metal oxide/silica surface from adsorption of chlorinated and hydroxylated benzenes.

Environmentally persistent free radicals (EPFRs) are deriving mostly from incomplete combustion of organic materials; they are typically formed on particulate matter through the interaction with aromatic hydrocarbons, catalyzed by transition-metal oxides, and produce reactive oxygen species (ROS) in aquatic media that may initiate oxidative stress in biological media.^1−3^ The generally accepted mechanism of EPFR formation involves chemisorption of chlorophenols and/or chlorobenzenes on the transition-metal oxide, Figure 1 (LSU model).^4^ This induces an electron transfer from the organic adsorbate to the transition-metal center, which results in the formation of the organic EPFR and in the reduction of the transition metal, Figure 1. The LSU model is well developed and widely accepted and utilized in various research centers worldwide^5,6^ (ref. Figure S1 in the Supporting Information).

The LSU model gets its roots from the tobacco research. As first reported in early 1958, cigarette tar has long-lasting paramagnetism.^7^ In one study of this paramagnetism, multiple radicals were identified in a tar and delocalized electron on a large PAH (polycyclic aromatic hydrocarbon) molecule.^8^

The seminal work on tobacco gas- and particulate-phase radicals was performed by Pryor and colleagues.^9,10^ For over 3 decades, beginning in 1970, Pryor and his colleagues have performed fundamental experiments in detecting, identifying, and isolating gas-phase and tar radicals formed from tobacco smoking.

More controlled studies of the formation of radicals from tobacco pyrolysis/oxidative pyrolysis have been performed by Dellinger and colleagues.^11−13^ It was hypothesized that some types of tobacco radicals are consistent with surface-associated, carbon-centered radicals, in which an unpaired electron is vicinal to an oxygen-containing functional group or a partially delocalized electron is associated with the bulk of a phenoxyl-type polymeric matrix.^11^

Dellinger and co-workers, in parallel with the tobacco research, widely developed and realized the formation of persistent free radicals (PFRs) as surface-bound radicals in the formation of dioxins,^14,15^ the formation of PFRs on metals/metal oxide surfaces^15^ (lately known as EPFRs), and the role of PFRs in the toxicity of airborne fine particulate matter, PM,^1,16^ as a new class of pollutants.^17^ The toxicological consequences of EPFRs are widely shown in numerous publications from LSU since 2000^1,16^ and the LSU Superfund Research Program (SRP) since 2009.^3,18−20^

The research by the LSU SRP on detection/identification of EPFRs was successfully extended and developed for environmental particulates PM2.5,^21^ contaminated soil and sediment samples,^22^ superfund soil samples,^23^ samples from plants’ phytometric measurements^24,25^—a new developing area of EPFRs, and EPFRs on engineered nanoparticles.^26^ In a recent publication,^27^ we have reviewed that the sources for EPFR formation range from automobile combustion engines, refineries, biomass in households, and waste incinerators; combustion systems have been shown to be a leading source of PM worldwide, with 70 to 90% of airborne PM originating in combustion processes.

It is worthy to note about substantial efforts of scientists from two laboratories working in parallel to Dellinger’s group on detection/identification of PFRs bound on PM in the early 2000s: Valavanidis and co-workers contributed much in recognizing of radicals bound to PM as persistent in environmental samples from combustion sources, combustion of common types of plastic, vehicular exhaust, incineration facilities, tobacco smoke, and so forth.^28,29^ Hopke and co-workers claimed that a wide range of ROSs appear in the gas phase of secondary organic aerosols (SOA) as very unstable intermediate products, such as hydrogen peroxide, organic peroxides, diacyl peroxide, peroxynitrite, and so on, and in the particulate phase.^30−32^

Other researchers also considered the ability of SOAs to generate ROS, especially •OH upon the interaction of SOA with water.^33,34^ A significant and a thorough research was reported recently about the particle-boundreactive oxygen species, PB-ROS, including neutral intermediate organics, among radicals associated with PM.^35^

### 1.1 Worldwide Development of EPFRs

The origin and nature of EPFRs, studied for a long time in Dellinger’s laboratory at LSU, were expanded and dispersed over many research laboratories worldwide.^5,6,36−39^ The number of studies related to EPFR chemistry and environmental and health impacts is constantly growing.^5^ EPFRs have emerged as an important PM component due to their special environmental and health effects.^1,18−20^ To illustrate the importance placed on these EPFR compounds by the research community and the society at large, it is interesting to note the explosion of the literature related to the topics of “EFPR” or “environmentally persistent free radicals” (Figure S1 from Web of Science, Supporting Information) in the past 5 decades, especially with the onset of the ground-breaking research initiated at LSU in the early 2000s.

A large amount of attention was paid to EPFRs formed on biochars^40−42^ and carbonaceous adsorbents.^43−45^ A comprehensive description of the formation, characteristics, and applications of surface-bound EPFRs in biochars is presented in refs (5) and (46).

### 1.2 Alternative Mechanisms for EPFR Formation

The more than decadal research about the formation and toxicological consequences of different EPFRs was based on the fundamental approach presented in Figure 1; the data generated from SRP at LSU were successively applied to obtain the results under lower temperature (<600 °C) conditions when the metal oxide surfaces were partially hydroxylated. However, with increasing temperature, the dehydroxylation of the metal oxide surface accelerates and the adsorption/chemisorption of the organic pollutants changes^47,48^ (ref. also the Supporting Information, Section 1).

A different pathway on the formation of EPFRs has been reported by D’Arienzo et al.;^37^ for reaction of benzene vapors and the CuO/SiO_2_ system, trace amounts of O_2_ were needed for the formation of the phenoxy persistent radical, whose stability was ascribed to the interaction with the oxide surface rather than the metal center (Supporting Information, Section 1). Chemosorbed benzyl radicals were generated on *metal-free* silica nanoparticles by Valeria B. Arce et al.^36^ (Supporting Information, Section 1).

In the current study, as an alternative to the metal-oxide-assisted formation of EPFRs described above and defined in the LSU model in Figure 1, we will present a metal-free generation of EPFRs (denoted here as bio-EPFRs) from pyrolysis one of the major components of biomass, lignin. The importance of the formation of EPFRs from pyrolysis of biomass burning has been highlighted recently by us^26^ and other researchers.^5^ Some initial results involving our recent findings on the formation of resonantly stabilized radicals from homogeneous pyrolysis of lignin and its monomers and precursors transferred into the gas phase by dispersion have also been reported.^49,50^

## 2 Experimental Section

### 2.1 Pyrolysis of Hydrolytic Lignin Dispersed into the Gas Phase; Continuous Atomization Reactor

**Figure 2:**
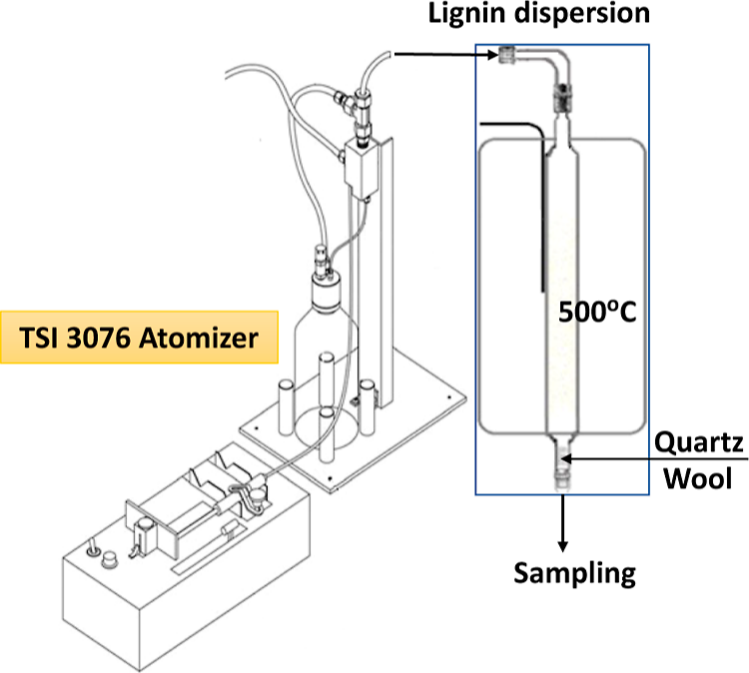
CA reactor coupled with a TSI 3076 atomizer.

To perform the pyrolysis of lignin in a continuous mode in the gas phase, hydrolytic lignin (HL) dark-brown powder (Sigma-Aldrich Inc., USA; detailed characteristics of HL is reported in the literature^51^) was dissolved initially in a solvent mixture of acetone/water (v/v of 9:1). The HL solution was introduced to the reactor using a commercial TSI 3076 Constant Output Atomizer; the reactor was denoted as continuous atomization (CA) reactor,^49,50^Figure 2. A flow of ultra-high-purity nitrogen gas was used to operate the atomizer and resulted in a 2 s residence time inside the quartz reactor (I.D. = 45 mm and *L* = 50 cm) situated in an electrical furnace. The pyrolysis products/radicals were trapped by deactivated quartz wool located at the end of the reactor, Figure 2. The quartz wool was transferred into electron paramagnetic resonance (EPR) tubes (o.d. = 10 mm), dried by flow of N_2_, and analyzed by EPR at room temperature.

The combination of different reactor sizes and carrier gas flow rates resulted in various residence times for pyrolysis reaction to occur in the gas phase. To measure the mass delivery rate of the atomizer, Cambridge filters were used as a trap to collect the dispersed lignin particles at the entrance and the exit of the reaction chamber.

The pyrolysis products were trapped by dichloromethane (DCM) in an impinger attached to the sampling port (Figure 2) at either the iced water or dry ice (−78.5 °C) temperature. The DCM solution containing all the trapped products was concentrated to ∼100 μL, and 1 μL was used for analytical analysis.

More details about the X-band EPR, high-frequency EPR (HF-EPR), and ESI-TOF-MS analysis are reported in the Section 2, Supporting Information.

## 3 Results and Discussion

### 3.1 Resonantly stabilized radicals from vacuum pyrolysis of lignin and model compounds

The importance of the radical mechanism of lignin pyrolysis as one of the major depolymerization pathways has been discussed in our recent publication.^49^ The respective free radicals have been detected in a variety of systems associated with pyrolysis of lignin and lignin model compounds, such as cinnamyl (CnA), *p*-coumaryl (*p*-CMA), and recently reported coniferyl (CFA) alcohols,^50,52,53^ using a low-temperature matrix isolation (LTMI) EPR technique.^54^ Detailed information about cryogenic trapping of radicals is presented in the Supporting Information (e.g., Figures S3 and S4 for some models and for lignin, respectively).

The high *g* values^a^ of the cryogenically trapped radicals have mostly been attributed to the dominant presence of different O-centered radicals (Table S1 and Figure S5) formed during primary, vacuum pyrolysis of monolignols^50,52,53,55,56^ as well as lignin itself^49^ (Figure S6a).

### 3.2 Resonantly Stabilized Radicals from Pyrolysis of Lignin Dispersed into the Gas Phase (CA Reactor)

**Figure 3:**
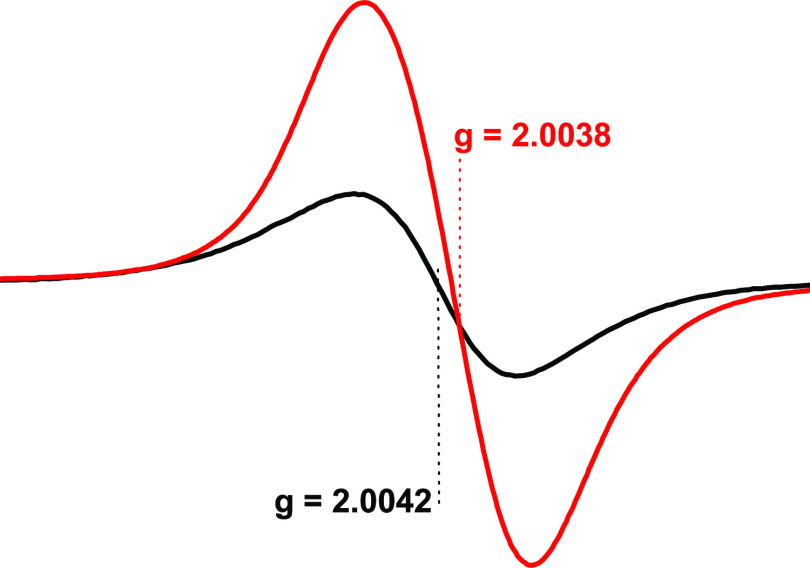
EPR spectra of intrinsic radicals from initial lignin (black line, *g* = 2.0042) and from pyrolysis of lignin dispersed in the gas phase, red line, *g* = 2.0038 at 9.76 GHz of the X-band EPR spectrometer.

Dramatically different results from those performed in vacuum were obtained from pyrolysis of lignin dispersed in the gas phase in a N_2_ carrier gas environment at 1 atm pressure in a CA reactor. The intermediate radicals were collected on deactivated quartz wool located at the end of the CA reactor and subjected to EPR analysis. A structureless singlet line with a narrow spectral width (Δ*H*p-p = 7 G) and low *g* values (below 2.0038) was observed (Figure 3) in contrast to the EPR spectra registered from vacuum pyrolysis of lignin (or model compounds), which consistently show high *g* values (Table S1). The radicals from lignin pyrolysis in the CA reactor (Figure 3, the red line) were surprisingly stable at room temperature (Section 3.3).

### 3.3 Lifetime of PFRs from Pyrolysis of HL Dispersed into the Gas Phase

**Figure 4:**
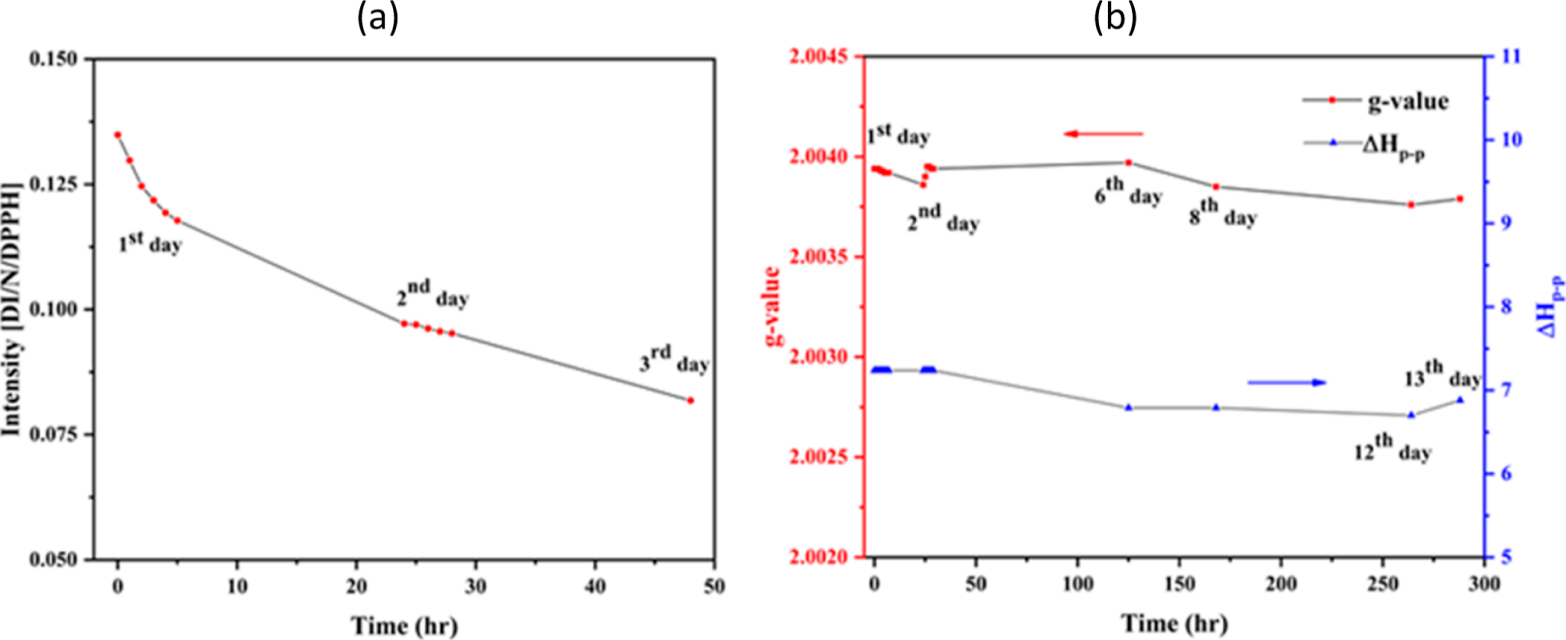
(a) Aging experiments for the PFRs from lignin pyrolysis; (b) Time dependence of *g*-values and Δ*H*p-p for PFRs. Practically no change in *g* values (∼2.0039), and Δ0Hp-p (∼7G) has been seen in the first 50 h. The experiment was performed in the CA reactor at 500 °C. The lignin initial solution concentration was 2 g/L, and the residence time was 2 s at an N_2_ gas flow rate of 3 L/min. Each experimental point was averaged from two different measurements.

To evaluate the persistence of the radicals (accumulated on the quartz wool in the CA reactor), the samples were subjected to EPR examination over long periods of time (aging), Figure 4 a. The lifetime of the PFRs is specified as a time (*t*) when the concentration of PFRs drops to 1/*e* times of its initial values (*e* is the natural logarithm base). For a pseudo-first-order decay reaction of EPFRs, the lifetime can be represented as **t**_**1/e**_ = **1/k**, where **k** is the decay rate constant determined experimentally using the standard equation below
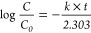
where *C*_o_ and *C* are the initial and current concentrations of EPFRs, respectively, and *k* is the pseudo-unimolecular reaction rate coefficient. The intensity of radicals was measured by EPR and normalized to DPPH standard.

The complexity of the decay process is illustrated by the aging profiles of involving decay of the two different groups of radicals. The first group with a 1/*e* lifetime of 33 h includes radicals rapidly decomposing during the first 10–12 h of measurements after accumulation on the quartz wool, whereas the second group with a 1/*e* lifetime of 143 h contains radicals that are more persistent and decomposing during 20–50 h of measurements after accumulation on quartz wool. Apparently, generation of such radicals (assigned here as bio-EPFRs) may have significant environmental/toxicological impacts yet to be assessed.

To further elucidate the complex nature of the radicals from gas-phase pyrolysis of HL in the CA reactor, a HF-EPR examination was initiated in cooperation with the National High Magnetic Field Laboratory, NHMFL, at Tallahassee.

### 3.4 HF-EPR Analysis of the Radical Mixture from Pyrolysis of HL in the CA Reactor

It is rather challenging to resolve the overlying EPR spectra (Figures S5 and S6) using a conventional X-band EPR spectrometer (operating at ∼9.52 GHz). However, some limitations can be largely compensated by using higher microwave frequencies/high magnetic fields, namely, via high-field EPR spectroscopy (HF-EPR).^57−59^ HF-EPR allows to increase the resolution of signals that overlap at lower frequencies—the higher the working frequency, the better the *g* tensor resolution.^57,60^ Using HF-EPR spectroscopy, it is possible to resolve the three principal components, *g*_*xx*_, *g*_*yy*_, and *g*_*zz*_, of the *g* tensor, as was recently demonstrated for radicals on natural humic acid by Christoforidis et al.^57^ They identified two π-type radical species presented in humic acid alike phenolic model compounds such as gallic acid.

Lignin isolated from biomass also contains a significant amount of stable organic radicals. The first attempt to resolve the nature of intrinsic radicals from lignin by itself has been performed only recently.^58^ The radical species were assumed to be semiquinone-type radicals stabilized in the polyphenolic lignin matrix.^58^ Using HF-EPR spectroscopy at 263 GHz, the authors were able to determine the *g*_*xx*_, *g*_*yy*_, and *g*_*zz*_ components of the *g* tensor of the stable organic radicals in raw lignin. With the enhanced resolution of HF-EPR, distinct radical species could be found in this complex polymer. The radical species were assigned to substituted *ortho*-semiquinone radicals and can exist in different protonation states.

While the radicals in lignin are closely related to those in humic acids, the corresponding data for radicals produced from lignin pyrolysis are still missing in the literature.

#### 3.4.1 HF-EPR Examination of Intermediate Radicals from Pyrolysis of Lignin Dispersed into the Gas Phase

As presented in this work and elsewhere,^61^ pyrolysis of lignin dispersed into the gas phase generates significantly higher concentrations of intermediate oligomer radicals compared to intrinsic ones presented in raw lignin, as shown in Figure 3. To perform HF-EPR examination of the radicals, the pyrolysis products were accumulated on deactivated quartz wool, transferred into PE plastic tubes (o.d. 8 mm, Fisher, 03-338-1A), packed tightly by slices of PTFE rods as plugs (1/4″ McMaster 8546K11) with the length less than 10 mm, and shipped to NHMFL at Tallahassee for HF-EPR analysis.

**Figure 5:**
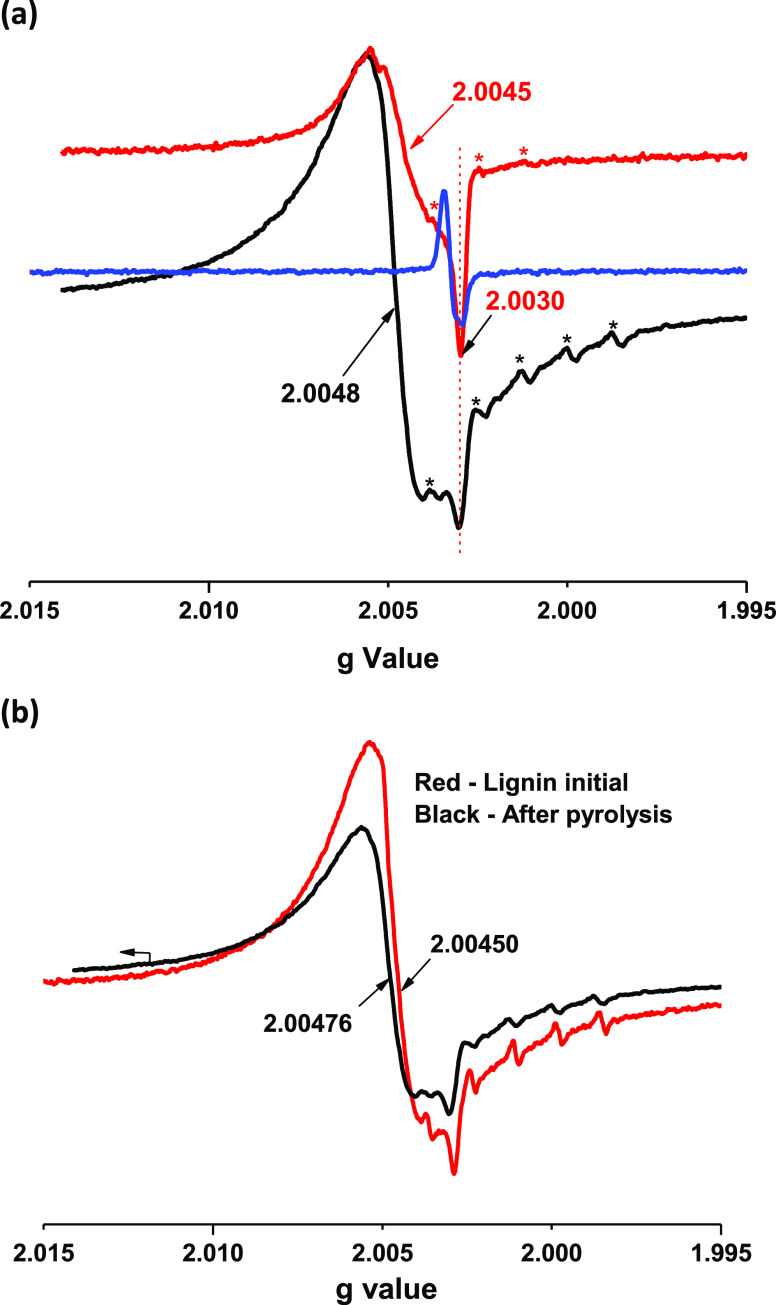
(a) HF-EPR spectra of radicals from lignin pyrolysis in the gas phase and detected at 5 K (red line) and 413 GHz. Black spectrum—the same sample detected at 20 K. The blue HF-EPR spectrum is for radicals from combustion reaction of 1-MN with additives (Supporting Information), detected at 5 K. The six-line patterns marked by asterisks are due to trace amounts of Mn^2+^. (b) Comparison of HF-EPR spectra of radicals from pyrolysis of lignin (black line) with the initial lignin (red line) detected at 5 K and 413 GHz.

The preliminary results demonstrated a substantial increase of the spectral resolution with increasing frequency of 9.52 GHz (X-band, Figure 3, red line, *g* = 2.0038, detected at room temperature) to 413 GHz (red spectrum in Figure 5a, *g* = 2.0045, detected at 5 K). While the spectrum is still not well resolved, a noticeable new component appears with a higher *g*_iso_ value of 2.0045 (at the crossing point of the baseline with the red spectrum).

Further interpretation of the red spectrum can be achieved by increasing the registration temperature from 5 to 20 K (or 40 K); the HF-EPR red spectrum converts to a black one now with a *g*_iso_ value of 2.0048 (Figure 5, a).

It appears that the spectrum with lower *g* values (below 2.0045—the refracted line in the red spectrum) was masked at temperatures higher than 5 K (20, 40 K). Note that the magnetic susceptibility of some magnetic species decreases while increasing the detection temperature above 5 K^62^ and this can be a reason for not detecting a component with a low *g* value at 20 K (40 K), Figure 5a, black spectrum. To clarify the nature of the black spectrum, it was compared with the initial lignin HF-EPR spectrum detected under the same conditions (Figure 5b, red line). Both spectra look like each other and resemble a neutral semiquinone-type radical with a high *g* value of 2.0044 indicated in ref (58).

Concerning the nature of the hidden radical (the refracted red line in Figure 5a) while increasing the detection temperature from 5 to 20 K, it can be hypothesized that it could be a carbon-centered radical with a *g* value between 2.0045 and 2.0030. To verify this assumption, we overlaid on the red spectrum an HF-EPR spectrum of radicals (Figure 5a, blue spectrum) derived from combustion of 1-MN (1-methylnaphtalene) including some additives (ref. Section 1, Supporting Information). Our prior research revealed that the radicals from combustion of 1-MN mostly consisted of carbon-like radicals [a mixture of soot radicals, radicals from polycyclic aromatic hydrocarbons (PAHs), C-centered organic radicals, etc.] with a *g* value of ∼2.0030.^63^ Similarly, the char from conventional pyrolysis of lignin (pyrolysis of lignin powder in the solid phase) at 500 °C also contains radicals resembling a soot (char) radical EPR spectrum with a *g* value of ∼2.0030.^49^ Apparently, additional experimental work is necessary to confirm the nature of radicals presented in Figure 5a, red spectrum. Note that the six-line patterns shown by asterisks in spectra Figure 5 are due to trace amounts of Mn^2+^ in lignin, which has been reported elsewhere^58^ and seems to be detected only by the HF-EPR technique.

A mechanistic interpretation and additional experimental confirmation for the formation of oligomer radicals from lignin pyrolysis in the CA reactor are presented below.

### 3.5 Oligomer, Resonantly Stabilized Radicals from Lignin Pyrolysis in the CA Reactor

**Scheme 1:**
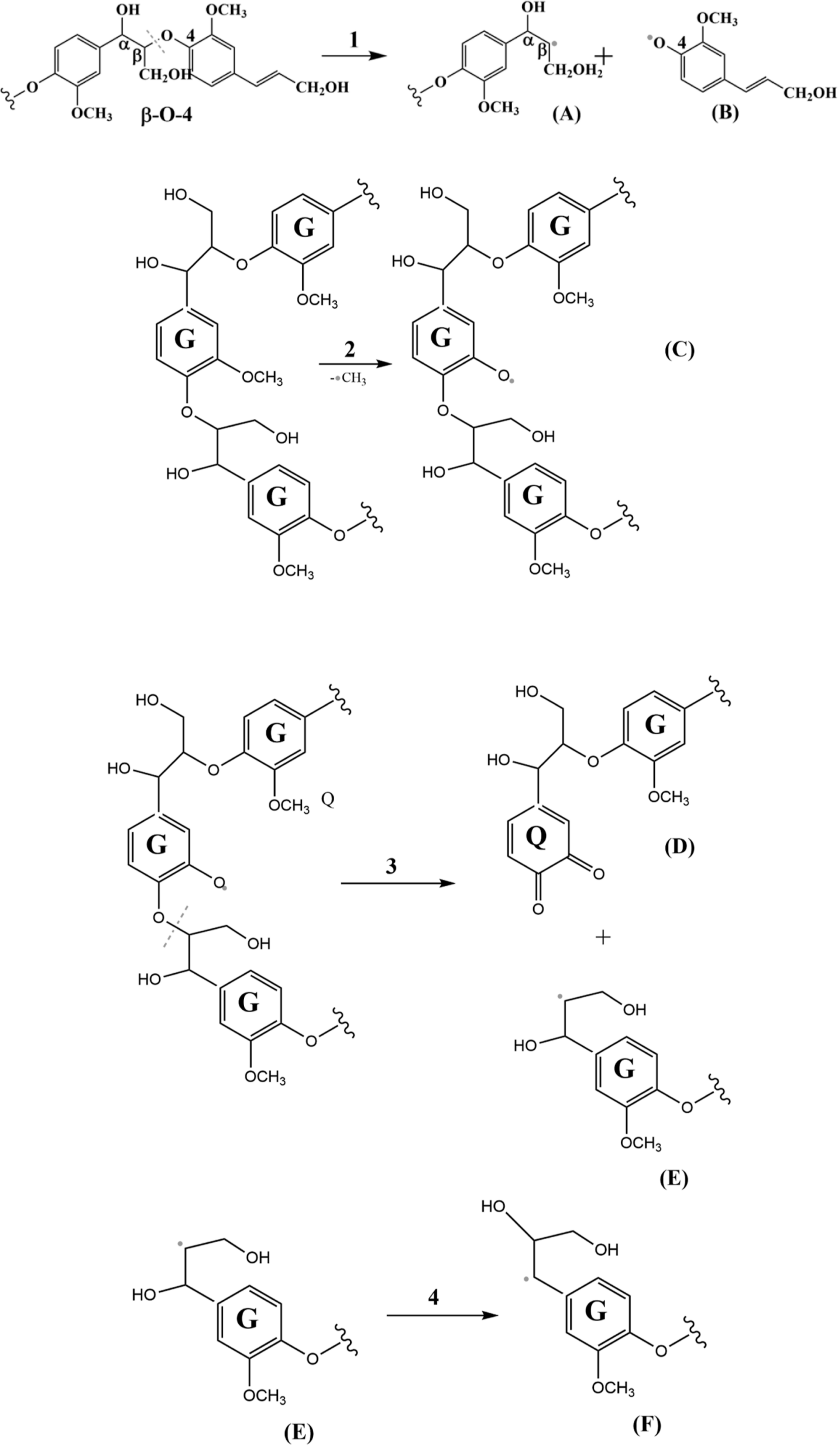
Schematic Representation for the Formation of Oxygen- and Carbon-Centered Radicals from Pyrolysis of “Bulk” (Reaction 1) and “End-wise” Type Lignin (Reactions 2, 3). Further Conversion of Radical (E) into Resonantly Stabilized Radical F Occurs in Reaction 4. **G** Stands for the Guaiacol Sub-unit

Scheme 1 (rxns 1–4) provides a mechanistic sketch of the radical formation pathways based on our understanding of the homogeneous gas-phase pyrolysis of lignin^49,61,64^ as well as current experimental results discussed above. Lignin is thought to be composed of well-defined structural moieties: so-called “bulk polymer” characterized by a large number of C_3_-side chains, Scheme 1 (reaction 1, left hand side), and the “end-wise” polymer, in which the β-O-4 linkage is dominant,^65^Scheme 1 (reaction 2, left hand side). In particular, the resonantly stabilized coniferyl phenoxy radical (B) can be generated from “bulk polymers” rich in C_3_ side chains, reaction 1.

On the other hand, a macro-phenoxy type radical (C) is generated following the initial demethylation of the “end-wise” lignin (reaction 2). A closed-shell oligomer (D) with the quinone end unit and a residue β-carbon-centered radical (E) are subsequently produced from intermediate (C) in the downstream reaction 3. Hence, the β-carbon-centered intermediate radicals (A) and (E) of similar structures can be formed from pyrolysis of both “bulk” and “end-wise” lignin, Scheme 1.

These types of radicals can also be formed from addition of OH pool radicals to the double bond of the monolignol side chains: (E) is the adduct of α-OH-addition, whereas (F) is formed via β-OH-addition. The reactivity of these species has been thoroughly explored theoretically by Asatryan et al., who suggested a multitude of unimolecular reaction pathways.^55^ The terminal O-centered radicals in turn are prone to dehydrogenation and elimination of formaldehyde from the C3 end chain, leading to the formation of oligomeric molecules.^56^ In addition, a shift of the OH group from the α- to β-carbon position, reaction 4, leads to interconversions of radicals. Due to the formation of the partially stabilized α-carbon-centered radical (reaction 4), the resonantly stabilized radical (F) encounters only 25 kcal/mol activation energy.^56^ Almost the same amount of energy is required for elimination of formaldehyde from the terminal O-centered radical.^56^

*Therefore, it can be safely concluded that the pyrolysis of either type of lignin produces resonantly stabilized C- and O-centered bulky radicals*. Because of the steric hindrance, however, the resonantly stabilized (C) and (F) polymer radicals may have less chance to participate in secondary reactions, thus reaching the sampling port of the CA reactor (Figure 3) and accumulating on quartz wool, in contrast to the more reactive smaller intermediate radicals that quench quickly. Thus, the bulky radicals are stable and detectable by EPR at room temperature. The oligomer radicals to some extent tumble at room temperature in the magnetic field by averaging *g*-tensor components and leading to the formation of an isotropic, close to symmetric, EPR line, Figure 3.

In fact, the residual spectrum in Figure 5b (black line) after the lignin pyrolysis *is a mixture of intrinsic radicals stabilized on unreacted lignin macromolecule(s) and newly formed radical-like C in*Scheme 1*or the oxygen-centered one in Scheme S1* (Section 6, Supporting Information). Perhaps, the newly formed O-centered radicals prevail in the mixture since the intensity of pyrolysis radicals (at 500 °C) is larger by a factor of 3 from the intensity of initial EPR spectrum of lignin, Figure 3 or Figure S10 (Section 7, Supporting Information).

The relative dominance of O- or C-centered radicals in a radical mixture depends mostly on the pyrolysis temperature, residence time, and the structure of precursors. The radicals with highly delocalized electronic structures with no functional groups attached to the carbon backbone possess a low *g* value, resembling a soot radical (a *g* value of around 2.0030^63^). The radicals with a localized high electron density in the vicinity of an oxygen atom possess a higher *g* value; for instance, the calculated spin density on the phenolic oxygen atom of the pure phenoxy radical (Figure S7a) is 0.41 at a *g* value of 2.0062, while it is much higher (*g* = 2.0220) for the radical located at the terminal oxygen atom (Figure S7b) with a spin density of 0.87, both calculated at the same UwB97XD/6-31+G(d,p) level of theory.

The O-centered radicals with a calculated high electron spin density and *g* value have been detected from vacuum pyrolysis of a number of lignin model compounds.^53,55^ Generally, as substituent groups appear at the aromatic ring and the spin-density is shifted toward the nearby oxygen center, the *g* value is also dropped, see, for example, the calculated *g* value for the (b) resonance structure of the phenoxy-type p-coumaryl radical in Figure S8. Therefore, the radicals with a low *g* value detected from HL gas-phase pyrolysis under atmospheric conditions, Table S1, can be represented by a radical with the spin density delocalized over the aromatic ring and the nearby functional groups [like bulky radicals (C), reaction 2 or (F), reaction 4] in Scheme 1 or just a large C-centered radical with localized electrons [like radicals (A), reaction 1 or (E), reaction 3]. These fragment radicals are mostly generated from primary breakdown of lignin macromolecules.

The secondary reactions of the reactive intermediate radicals can further contribute to their steady-state concentration depending on the stability and reactivity of these species.

### 3.6 Fate of the Monomer Radical (B) Generated during Lignin Pyrolysis—Reaction 1 in Scheme 1

**Scheme 2:**
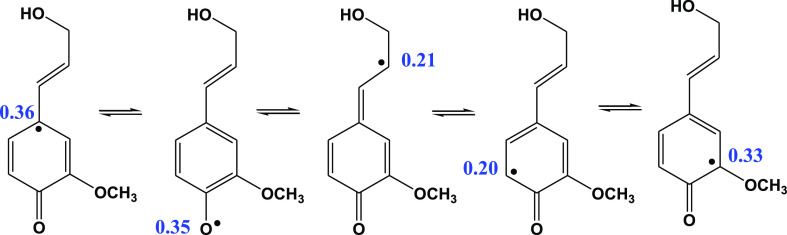
Mesomere Forms of the Gauche-Phenoxy-Type Radical of CFA Numbers represent spin densities calculated at the M06-2X/6-31+G(d,p) level of theory

The LTMI EPR data from low-pressure vacuum pyrolysis of coniferyl alcohol (CFA) reveal the formation of various types of resonantly stabilized carbon- and O-centered radicals.^50^ The spin distribution of the mesomere (resonance) forms of the phenoxy-type radical of the CFA is presented in Scheme 2. This radical, similar to other phenoxy-type monolignol radicals, serves as a precursor species for lignin synthesis.^66^ It is believed that it undergoes coupling at various sites to form different interunit C–O and C–C bonds.^67,68^ The oligomerization phenomenon has been advocated by Smith and Lee based on petroleomic analysis of bio-oil derived from biomass fast pyrolysis.^51^

To elucidate the critical role of CFA alcohol radical (B), reaction 1 (Scheme 1) in the process of lignin pyrolysis, a separate pyrolysis experiment was performed involving a model compound, CFA in the same CA reactor at a residence time of 2 s (Supporting Information, Section 6). ESI (electron spray ionization) MS analysis of products from CFA pyrolysis in the CA reactor revealed the formation of oligomer compounds with the molecular masses up to 550 amu (Figure S9, Supporting Information). It demonstrates that the radicals from CFA pyrolysis (Scheme 2), particularly the (B)-type resonantly stabilized persistent radicals from reaction 1, are polymerized in the gas phase to produce dimers,^68^ trimers (Supporting Information, Section 6, Scheme S1), and so on. Indeed, the calculated lifetime of the O-centered radicals of CFA (CFA_oxy radical_) based on their interaction with CFA molecules is in the order of 1 s, which is sufficient for their dimerization in the CA reactor at a residence time of 2 s (ref. Supporting Information, Section 6).

In summary, persistent oligomer oxygen- or carbon-centered bulky radicals (bio-EPFRs) can be formed not only from direct fragmentation of lignin during pyrolysis (radicals assigned C, F—Scheme 1) but also because of the secondary reactions involving monomer radicals like (B) (for instance, via an oligomerization reaction shown in Scheme S1, Section 6, Supporting Information).

## 4 Conclusions

Persistent macroradicals are generated homogeneously in a metal-free environment from gas-phase pyrolysis of lignin dispersed into the nitrogen stream. We assigned these types of radical bio-EPFRs derived from pyrolysis one of the major components of the biomass lignin. The EPR spectrum of pyrolysis radicals collected on the deactivated quartz wool located at the end of the gas-phase reactor is represented by structureless, singlet lines with some anisotropy at *g*_iso_ ∼ 2.0033, which is changed depending on pyrolysis conditions up to 2.0040 using regular 9.52 GHz EPR measurements. Our research shows that the radicals from post-pyrolysis of HL constitute paramagnetic fragments of the decomposed lignin macromolecules and represent newly formed polymerized units containing both oxygen and carbon centers. Even though we did not yet achieve sufficient resolution of the EPR spectra of radicals using HF-EPR at 413 GHz, the initial experiments strongly support the existence of the O-centered radicals in the radical mixtures possessing a high apparent *g* value of 2.0048. These findings are in line with mechanistic interpretation of the results provided in this article.

## Supporting Information Available

Alternative mechanisms for EPFR formation; cold finger assembly with the pyrolysis reactor; electron spin distribution in radical mesomere structures; ESI MS analysis (PDF)

## Footnotes

a The ***g*value** measures how the magnetic environment of unpaired electrons differs from that of a free, gas-phase electron, *g* = 2.0023.
