# Supplementary material for: Metal-Free Biomass-Derived Environmentally Persistent Free Radicals (Bio-EPFRs) from Lignin Pyrolysis
Source: ACS Omega. 2022 Aug 16;7(34):30241–9. doi: 10.1021/acsomega.2c03381 (PMC9434622; doi:10.1021/acsomega.2c03381)
Supplement: Supplementary file 1 — ao2c03381_si_001.pdf [file ao2c03381_si_001.pdf]

Supporting Information

Metal-free Biomass Derived Environmentally Persistent Free Radicals (Bio-EPFRs) from Lignin Pyrolysis

Lavrent Khachatryan, [‡*] Mohamad Barekati-Goudarzi,[‡] Rubik Asatryan[l*], Andrew Ozarowski[+], Dorin Boldor[§], Slawomir M Lomnicki, [£], Stephania A. Cormier, [!]

[‡] Department of Chemistry, Louisiana State University, Baton Rouge, Louisiana 70803, United States.

[l] Department of Chemical and Biological Engineering, University at Buffalo, The State University of New York, Buffalo, New York, 14260, United States

[+] National High Magnetic Field Laboratory, 1800 East Paul Dirac Drive, Florida, Tallahassee, 32310, United States

[§] Department of Biological and Agricultural Engineering, LSU AgCenter and LSU A&M College, Baton Rouge, Louisiana, 70803, United States

[£] Department of Environmental Sciences, Louisiana State University, Baton Rouge, Louisiana 70803, United States

[!] Department of Biological Sciences, LSU Superfund Research Program and Pennington Biomedical Research Center, Baton Rouge, Louisiana 70808, United States

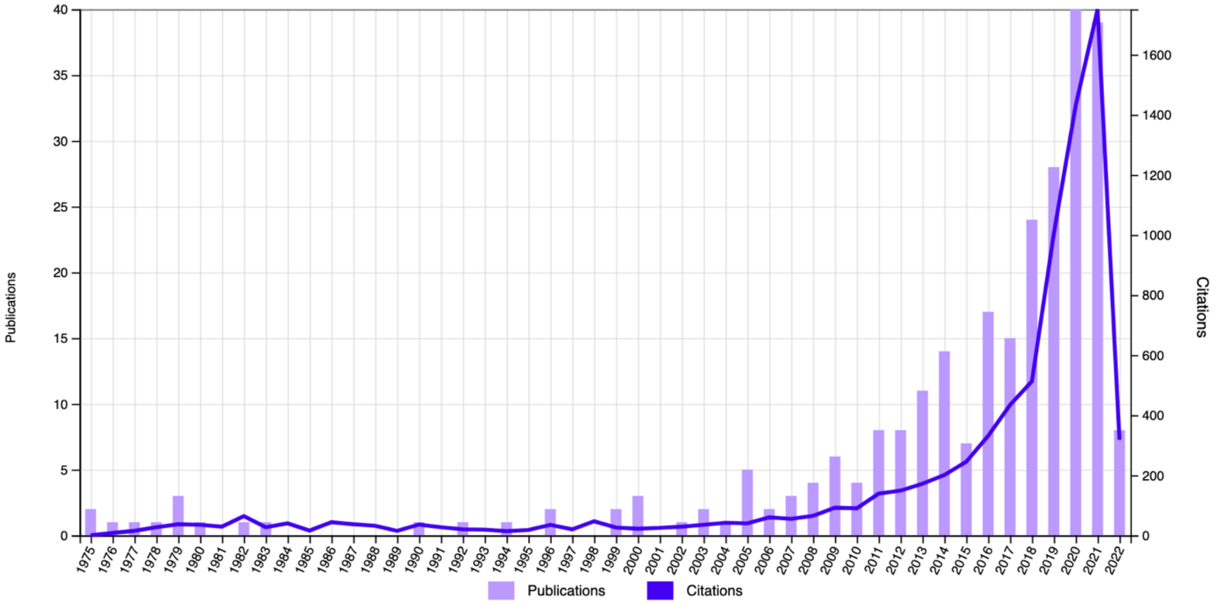

Figure S1. The number of publications and citations related to EPFRs available in Web of Science database (February 2022).

1. Alternative mechanisms for EPFRs formation (Literature data)

Formation of EPFRs on dehydroxylated metal oxide surfaces: Toluene has been reported to interact with metal and metal oxide surfaces by a charge transfer from the π orbitals of the aromatic ring to the metal center for dehydroxylated metal oxide surfaces, suggesting a parallel orientation of the reacting molecule with the surface [1-3]. The adsorption takes place through the formation of a π-complex between the surface Fe^{3+} ion and the aromatic ring. In the case of toluene, owing to the electron-donating nature of the CH_3 group, the increased electron density of the aromatic ring may be favorable for an even stronger interaction with the surface metal site [4].

These interactions lead to the destabilization of the aromatic ring and substituent groups and result in the surface-mediated hydrogen abstraction [2, 3]. The generated benzyl radicals may react directly or convert to more stable radicals, oxygenated species, PAHs, and soot particles [1-4]. In support of this theory, studies have shown that the surface-active centers of Fe_2O_3 nanoparticles undergo a reduction while the core composition remains unchanged under high soot formation conditions in a laminar ethylene-air diffusion flame seeded with iron pentacarbonyl [5].

A similar mechanism for partially dehydroxylated metal oxide surfaces has been implemented by Dellinger and co-workers for 1-methyl naphthalene combustion in presence of Fe(III) oxide as a catalyst at relatively low temperatures (<1100°C) [6]. It was suggested that a combined mechanism is responsible for the partially dehydroxylated Fe(III) oxide surface, i.e. standard interaction in the first stage (by analogy to the LSU model, Figure 1 in the main text) with hydroxyl groups on the surface (Path A) and electron transfer from the organic sorbate to the metal to form an organic EPFR (Path B) [6].

The EPFRs formed by oxidative pyrolysis/combustion of a forementioned organics were generated in two-stage combustion reactor and collected on Cab-o-Sil matrix at the exit of the reactor via thermophoresis technique. A typical EPR spectra of the samples are characterized by broad (ΔHp-p ~6 -10 Gauss), asymmetric signal with g value in the range 2.0028-2.0036. Our earlier research showed that the predominant species in radical mixture from combustion of 1-MN are soot like particulates with g values ~2.0030 [7]. Similarly, the char from conventional pyrolysis of lignin (pyrolysis of lignin powder in solid phase) at 500°C also resembles a soot radical EPR spectrum with g value ~ 2.0030[8]

The role of traces of oxygen in the formation of EPFRs: It has been shown [9] that copper(II) centers of copper oxide undergo partial reduction to Cu(I) during the vacuum annealing before interacting with the organic contaminants, and that benzene oxidation involves reduction of Cu(II) to Cu(I) centers. This hypothesis has been experimentally and theoretically confirmed by D'Arienzo et al. [10] demonstrating a distinct mechanism for formation of EPFRs from exposure of CuxO/SiO2 by

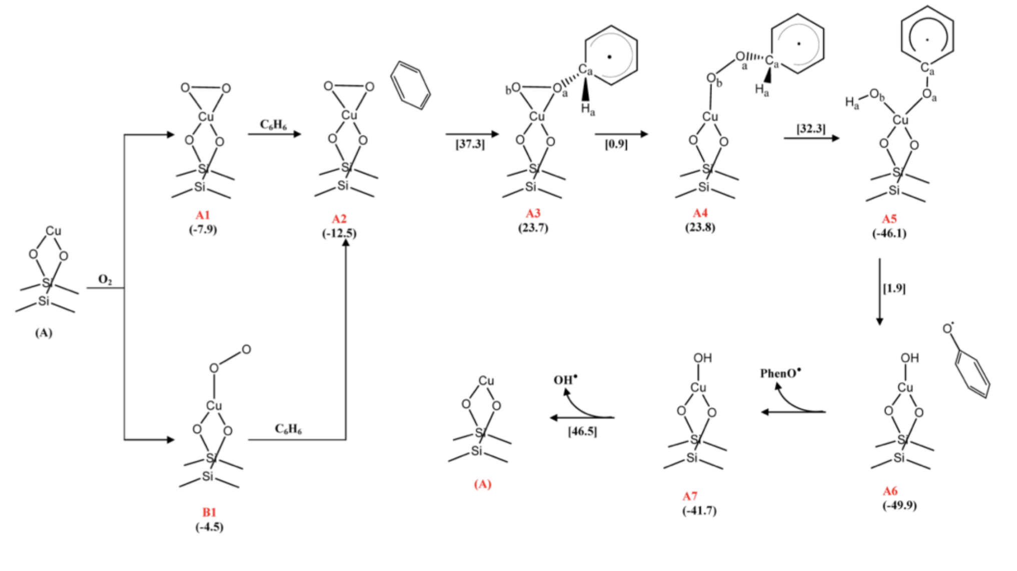

Figure S2. Catalytic Generation of Environmentally Persistent Free Radicals from Benzene.

saturated vapor of benzene, Figure S2; EPR spectroscopy revealed the formation of phenoxy radical entrapped in the catalyst upon reaction between benzene and CuxO/SiO_2. The results were discussed thoroughly in our previous publication [11]. We highlight, here, the great influence of O_2 on formation of EPFRs; trace amounts of O_2 needed for formation of phenoxy persistent radical whose stability was ascribed to the interaction with the oxide surface rather than the metal center [10]. The computational approach, represented by both a cluster and a periodic surface model of Cu(I) centers, allowed one to propose a reaction mechanism in which Cu(I) promotes the activation of molecular oxygen (receiving electrons from Cu(I)) by forming a Cu(II) -O2- complex. These species, reacting with benzene, yield a Cu(II)-phenoxy complex that, upon an essentially barrier-less dissociation, gives a phenoxy radical stabilized by interaction with the catalyst, Figure S2. In the last step, dissociation of the ●OH recovers the catalyst, with concomitant reduction of Cu(II) to Cu(I).

Immobilized benzyl EPFRs: Chemosorbed benzyl radicals were generated on *metal free* silica nanoparticles [12]. Functionalized silica nanoparticles (NP) were obtained by esterification of the silanol groups of fumed silica nanoparticles with benzyl alcohol via following reaction.

$$\geqslant \text{Si-OH} + \text{ROH} \rightarrow \quad \geqslant \text{Si-OR} + H_2O$$

where ROH is $C_6H_5CH_2OH$, benzyl alcohol. These particles were characterized by several techniques and used in acetonitrile ⁄ water suspensions in the presence of benzophenone (BP) to study the generation of surface-grafted radicals by excited states in solution and their reactivity. For that purpose, the decay kinetics of triplet BP in time-resolved phosphorescence experiments were investigated. The formation of organic radicals **C_6H_5—(•)CH—O—Si≡** attached to the nanoparticles surface was studied by laser flash-photolysis (LFP). The authors concluded that

immobilization of benzyl radicals covalently attached to silica surfaces led to enhancement of unimolecular radical decay pathways relative to bimolecular ones.

Note that the long-lifetime benzyl radicals adsorbed on *zeolites* were also reported to be avoiding the radical-radical re-combination, which typically constitutes the main decay route in non-viscous solvents [13]. The results obtained in the paper demonstrated that H-abstraction from methylene groups of benzyl alcohol chemisorbed on the silica nanoparticles leads to the formation of long-lifetime benzyl radicals covalently attached to the silica surface. However, the further persistence/reactivity of immobilized benzyl radicals detected in ref. [12] was not reported.

2. Experimental

EPR analysis: The X-band EPR spectra were recorded on a Bruker EMX-20/2.7 EPR spectrometer (Bruker Instruments, Billerica MA) with dual cavities, X-band (100 kHz) and microwave frequency at 9.76 GHz. The typical parameters were sweep width of 100 G, EPR microwave power from 1 to 64 mW, receiver gain of 10^4, modulation amplitude ≤4 G and frequency of 100 kHz, time constant and sweep time of 10.24 ms and 167.77 s, respectively

High Frequency (HF) EPR: The high-field EPR spectra at maximum frequency of 413 GHz were recorded at the EMR facility of the National High Magnetic Field Laboratory, NHMFL (https://national maglab.org/user-facilities/emr) using a home-built spectrometer. The instrument is a transmission-type device, using no resonance cavity, in which waves are propagated in cylindrical light pipes. The microwaves were generated by a phase-locked oscillator (Virginia Diodes) operating at a frequency of 8–20 GHz. This frequency was transmitted to a chain of frequency multipliers generating the 2nd, 4th, 8th, 16th, 24th, 32nd and 48th harmonics. The frequency is accurate to better than 7 significant digits. A superconducting magnet (Oxford Instruments) capable of reaching a field of 17 T was employed

The modulation frequency of 50 kHz was used in concert with amplitudes of 5 and 10 G, but these amplitude values are approximate due to the instrument limitation. Atomic hydrogen trapped in the octaisobutylsilsesquioxane nanocage was used as the g-factor standard for the high-frequency EPR measurements[14].

Magnetic measurements were conducted using a Quantum Design SQUID-based MPMSXL-5 magnetometer equipped with a superconducting magnet. The magnetometer was calibrated with a palladium rod sample (Materials Research Corporation, measured purity 99.9985%). The magnetic susceptibility data of a powder sample was measured over the temperature range of 1.8–300 K at a magnetic induction of 5000 G.

ESI-TOF-MS analysis

High Performance liquid chromatography (HPLC) coupled to accurate mass electrospray ionization (ESI) mass spectrometry was utilized in this analysis. Specifically, an Agilent 1260 Infinity II quaternary liquid chromatograph coupled to an Agilent 6230 Electrospray Time-of-Flight mass spectrometer was used for detection of analytes. The HL pyrolysis samples were dissolved in acetonitrile/water with 5% formic acid and run in positive mode ionization with a capillary voltage of 4000v. Drying gas (nitrogen) temperature was 325°C delivered at 10 l/min and the fragmentor voltage was set to 150 v. No LC column was used for sample delivery; only flow through injection was utilized (direct injection from LC to mass spectrometer). Mobile phases used were A: 30% LCMS grade water with 0.1% formic acid and B: 70% LCMS grade acetonitrile with 0.1% formic acid with a flow rate of 0.4ml/min

3.Cold Finger assembly

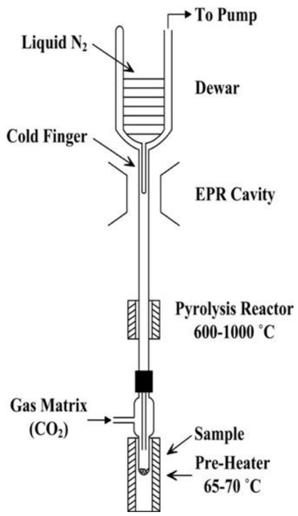

Figure S3 Vacuum pyrolysis of lignin model volatile compounds in conjunction with cold finger

The cold finger assembly is the central unit in LTMI EPR for cryogenic trapping of intermediate radicals produced during vacuum pyrolysis of any organic; the CO2 carrier gas enters in special container with solid(liquid) sample, flashes the vapor and directs to the cold finger under continuous pumping and at total pressure less than 0.3 tor. The CO2 gas is a good matrix at condensation of intermediates from pyrolysis and dilutes the radicals concentration on cold finger avoiding their mutual recombination [15].

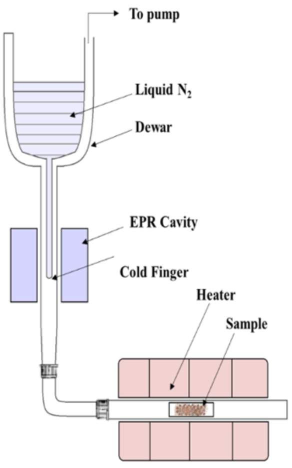

Figure S4. Vacuum pyrolysis of solid samples (particularly, lignin) in conjunction with cold finger.

The temperature in the heater for lignin pyrolysis was changed from 400 to 550oC[8]

Table S1. Comparison of g values of radicals produced from vacuum pyrolysis of lignin model compounds and HL lignin at different conditions

Compound	Phase	Pyrolysis conditions	g-values (Temperature, oC)	Comment
Cinnamyl alcohol	liquid	vacuum	**2.0085** (500); **2.0097** (800)	(a)
p-coumaryl alcohol	solid	vacuum	**2.0089** (700); **2.0102** (1000)	(a)
Coniferyl alcohol	solid	vacuum	**2.0125** (400); **2.0117** (500); **2.0114** (600)	(a)
Hydrolytic Lignin	Solid	Vacuum	**2.0080** (425- 525)	(a)
Hydrolytic Lignin	Dispersed*	In 1 atm. N2, gas phase	**2.0039** (490); **2.0037**(550); **2.0033** (700)	(b)

(a) - Detected at liq.N2 temperature; (b) – biochar from pyrolysis in CA reactor, detected at room temperature;
* Lignin dissolved in acetone:water = 9:1 solution and dispersed in CA isothermal reactor. Intermediate products and radicals were collected on quartz wool located at the end of the reactor.

4.Summary from cryogenic trapping experiments [8, 16, 17]

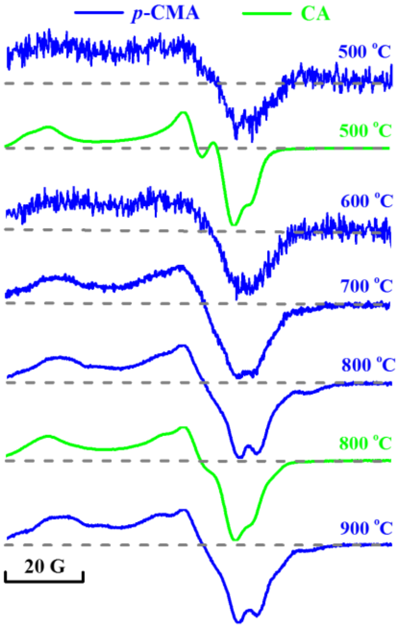

Figure S5. Comparison of the EPR signals from vacuum pyrolysis of p-CMA and CnA showing high g-values (2.0085-2.0095) that increased largely with temperature. The EPR spectra were detected at 77K using LTMI technique.

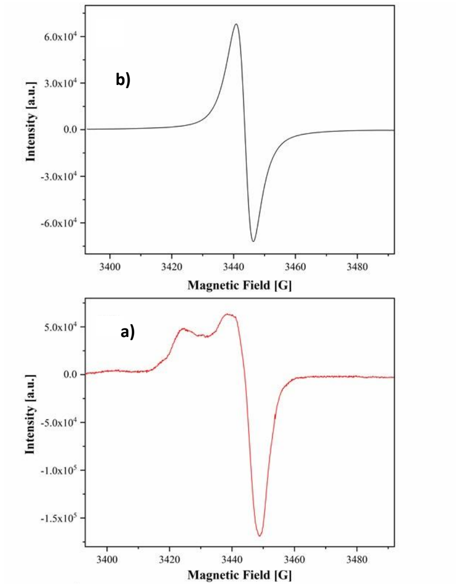

Figure S6. EPR spectra from pyrolysis of HL: **a)** EPR spectra of the radicals from vacuum pyrolysis of lignin (g-value of 2.0098); the EPR spectra were detected at 77K using LTMI technique. **b)** The residue accumulated on quartz wool from CA reactor at 500°C (g-value of 2.0037).

5.Type of radicals (via Electron spin distribution) vs. g values

(a) **(b)**

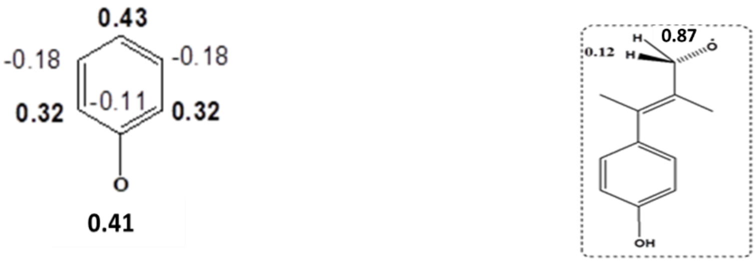

g_{iso} = 2.0062 g_{iso} = 2.0220

Figure S7. The spin distribution in (a) - phenoxy radical, (b) - localized terminal O-centered radical of p-CMA alcohol, calculated at B3LYP/6-31G** level for comparison showing dependence of g_{iso} on spin densities located on the **p^z** orbitals of the corresponding oxygen atoms.

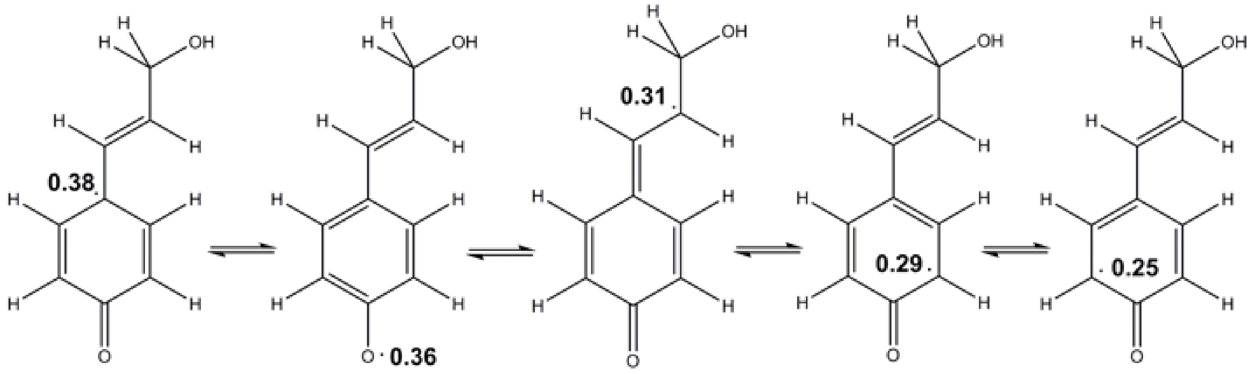

(a) **(b)** **(c)** **(d)** **(e)**

g iso = 2.0052

Figure S8. Highly delocalized phenoxy type p- coumaryl radical, (b), calculated at the uB3LYP/6-31G** level of theory. Numbers are electron spin densities presented for C-centered mesomere (resonance) forms (a), (c), (d) and (e) separately; the negative spins are omitted

6. ESI (Electron Spray Ionization) MS analysis of products from CFA pyrolysis in CA reactor.

A typical ESI MS spectrum registered before and after pyrolysis of CFA is provided in Figure S9. The existence of two strong characteristic peaks at 163 and 131 m/z from both before and after pyrolysis of CFA proves the incomplete conversion of CFA at 400oC. Previously, these peaks were assigned to the precursor ion $(CFA\text{-}H_2O+H)^+$ and product ion $(CFA\text{-}H_2O\text{-}CH_3\text{-}OH+H)^+$,

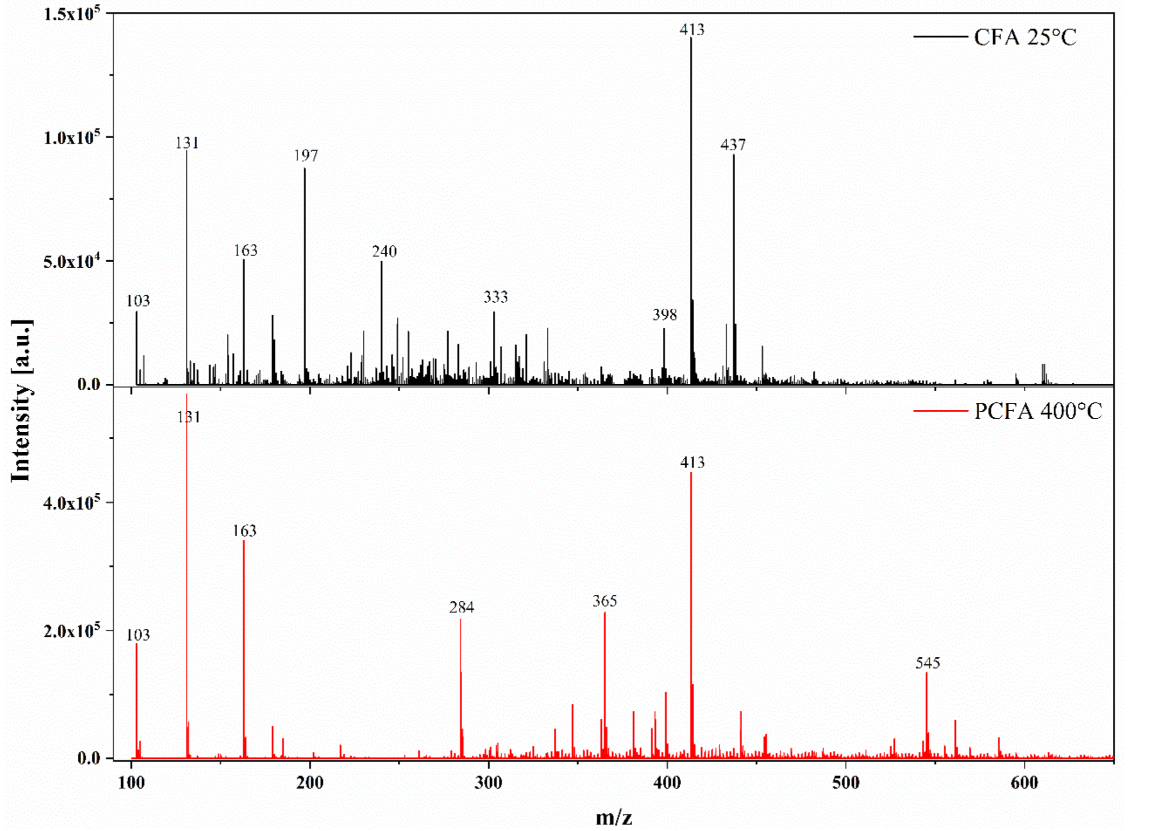

Figure S9. ESI-TOF-MS spectra of initial CFA dissolved (top spectrum) in acetonitrile and pyrolyzed CFA (PCFA, bottom spectrum) in CA reactor at 400°C.

respectively, formed from water loss of the electrospray ionization source. Also note the presence of some oligomeric forms (probably dimeric contaminants) from ESI analysis of initial CFA, Figure S9 top spectrum.

The analysis of ESI spectra shows some redistribution of products in the characteristic for dimers region (from 200 to 400 amu) and appearance of the traces of oligomers around 500 amu (ref. [18]); no major high molecular weight peaks (1000 and higher) were seen. This may suggest that the radicals from CFA pyrolysis (Scheme 2 in the main text) are polymerized in the gas phase to produce either quinone type intermediates, and/or stable oligomer radicals, such as a trimer O-centered radical provided in Scheme S1.

The major operational difference of this study in comparison to literature is the fluidization of CFA in gas phase which minimizes the particle size into extremely fine aerosol (<0.3 μm) using atomization technique as well as the elimination of reactive char surface contacts with the volatile compounds.

The easy condensation of CFA in conventional reactors most likely occurs only in the

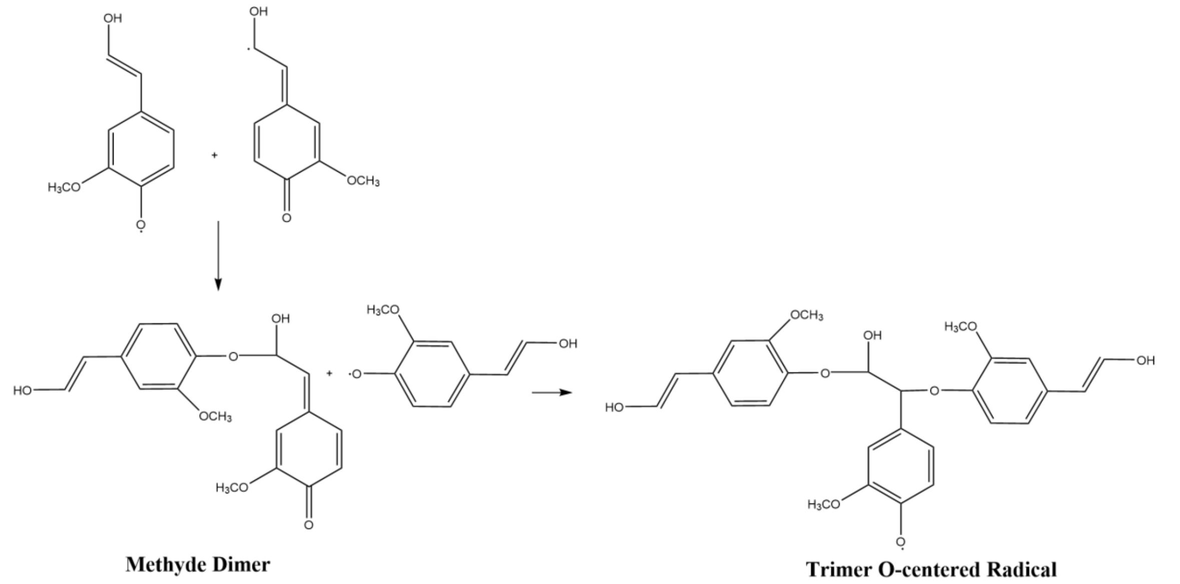

Scheme S1. Formation of quinone methide dimer through CFA radical-radical dimerization and further re-aromatization through radical +dimer molecule reaction by formation of O-centered resonantly stabilized oligomer radical

presence of particle surfaces, and the elimination of the intermediate interphase and solid char surfaces prevents further condensation of CFA during gas-phase pyrolysis.

The kinetic control of the condensation (coupling) reactions is important to understand formation of lignin intermediate macromolecules[19]. In this respect, valuable data concerning the activation energies of these coupling reactions are reported in ref. [19], Scheme S2. The weak interactions between reactants have been found to play an important role. The hydrogen bond formed between the oxygen of the phenoxy type radical, and the alcohol of the aliphatic chain, together with the interaction between aromatic rings, locates the reactants in a position that favors such β–O–4 linkage. It was theoretically calculated activation energies of the possible reactions for formation of dimeric units through the reactions radical + molecule, reaction (1) and radical + radical, reaction (2) using DFT theory, Scheme S2. Surprisingly, high activation energy of 16.7 and 15.9 kcal/mol were found for the reaction (1) and a radical-radical coupling reaction (2).

$CFA_{\textbf{oxy radical}}$ +p-CMA $_{\textbf{molecule}}$ → dimer radical reaction (1)

$CFA_{\textbf{oxy radical}}$ +p-CMA $_{\textbf{β carbon radical}}$ → neutral dimer reaction (2)

Scheme S2. Two possible reactions proposed for the formation of β–O–4 linkage in lignin; radical-molecule (1) and radical-radical (2) interactions.

It should be emphasized, however, that the authors of ref 19 could not locate any TS to follow reaction (2) - to generate a β-O-4 linkage; instead, they found a TS that leads to the (ortho/para) inter-ring coupling of the two radicals with Ea=15.9 kcal/mol. This phenomenon is consonant with the general dimerization mechanism of the two unsubstituted phenoxy radicals, which occurs through ring addition[21], and may suggest that the coupling of phenoxy-type monolignole radicals also are governed by the same types of interring interactions rather than interplay of conjugated side-chains. These very important calculations illustrate kinetic difficulties for condensation of monolignol radicals not only because of the high activation energies but also due to the steric hindrance requiring optimal orientation of interacting species (radical - radical, or radical-molecule) through weak intermolecular forces [19]. Indeed, the addition of the simplest

radical OH to the analogous beta-unsaturated carbon center of the monolignols, as a simplest analogue of the radical-molecule reaction (1), occurs without any activation barrier[22].

Therefore, we focused here on the formation of a dimer through a reaction similar to reaction (1), Scheme S2, which occurs between an open-shell CFA-radical ($CFA_{\textbf{oxy-radical}}$) and a closed-shell CFA molecule ($CFA_{\textbf{molecule}}$) relevant to the gas - phase processes in CA reactor:

$CFA_{\textbf{oxy radical}} + CFA_{\textbf{molecule}} \rightarrow$ dimer radical reaction (3)

The life time of $CFA_{\textbf{oxy radical}}$ (τ) with respect to the bimolecular reaction (3), is determined using the equation below,

$\boldsymbol{\tau = 1 / k_3 \text{ (CFA)}}$

where k_3 is the rate constant for the reaction 3) and (CFA) – the current concentration of coniferyl alcohol in the gas phase

The experiments were performed at low concentration of CFA; *ca.* 5.56 ug CFA is passed through the isothermal zone (1/3rd of the reactor) at each 2 sec passes, which corresponds to concentration of CFA *ca.* $2.67.10^{17}$ particle/cm3. A similar value of 16.7kcal/mol to the activation energy of the reaction (1) [19] can be employed for the reaction (3). Since this type of reaction needs strong geometrical orientation [19] the pre-exponential factor could be much less than 10^{-10} cm3/mole/sec typical for a bimolecular radical-molecule reaction[20]. A 10^{-13} cm3/mole/sec is reported for bimolecular reactions of radical+molecule[19]. With a bias toward the reaction (3) at $k_3 = 10^{-13}$ exp(-15.7kcal/mol/RT) cm3/mole/sec (the activation energy lowered by 1 kcal/mol since additional OCH_3 groups activate the reactivity of lignols), the calculated lifetime of CFA radical will be *ca.* 1sec using the above equation $\tau = 1 / k_3$ (CFA). This time is sufficient for the radicals' secondary reactions in CA reactor (Figure 2 in the main text) operating at residence time of 2 sec.

7. Increasing of pyrolysis temperature largely effects radicals' intensity during gas phase pyrolysis of lignin [17].

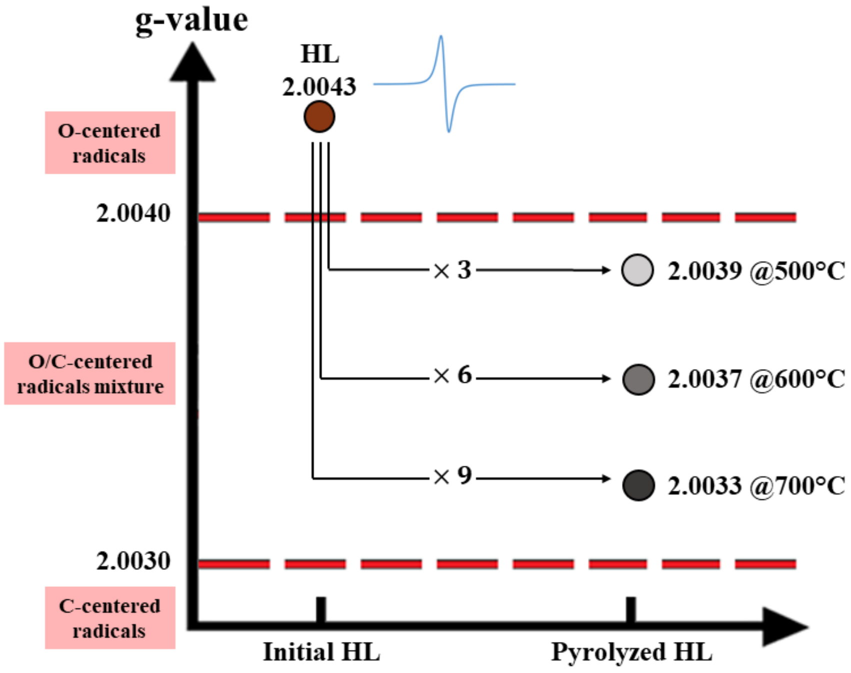

Figure S10. The trend of g-value of radicals accumulated on quartz wool located at the end of the reactor during homogeneous gas phase pyrolysis of lignin in CA reactor. The g-values 2.0030 and 2.0040 on Y axis are arbitrary marker values for C-, O- centered radicals, respectively, as well as a mixture of these radicals between these values. The increase in radical concentration is shown on each corresponding arrow for each temperature

References

1. Haber, J.; Tokarz, R.; Witko, M. Quantum-chemical description of the oxidation of alkylaromatic molecules on vanadium oxide catalysts. In New Developments in Selective Oxidation, Stud. Surf. Sci. Catal., Vol. 82; Eds. V. Cortes Corberan, S. Vic Bellon; Elsevier, Amsterdam, **1994;** pp. 739–748.
2. Irigoyen, B.; Juan, A.; Larrondo, S.; Amadeo, N., Adsorption reactions of toluene on the (110) vanadium antimonate oxide surface. *J.Catal.* **2001,** *201* (2), 169-182.
3. Irigoyen, B.; Juan, A.; Larrondo, S.; Amadeo, N., The adsorption of toluene on V-Sb oxides. Theoretical aspects. *Surf.Sci.* **2003,** *523* (3), 252-266.
4. Nagao, M.; Suda, Y., Adsorption of Benzene, Toluene, and Chlorobenzene on Titanium-Dioxide. *Langmuir* **1989,** *5* (1), 42-47.
5. Jasinski, J.; Pinkerton, K. E.; Kennedy, I. M.; Leppert, V. J., Surface oxidation state of combustion-synthesized gamma-Fe2O3 nanoparticles determined by electron energy loss spectroscopy in the transmission electron microscope. *Sens. Actuators, B.* **2005,** *109* (1), 19-23.
6. Herring, M. P.; Potter, P. M.; Wu, H.; Lomnikci, S.; Dellinger, B., Fe_2O_3 Nanoparticle Mediated Molecular Growth and Soot Inception from the Oxidative Pyrolysis of 1-Methylnaphthalene. *Proc. Combust. Inst..* **2013,** *34*, 1749-1757.
7. Herring, P.; Khachatryan, L.; Lomnicki, S.; Dellinger, B., Paramagnetic centers in particulate formed from the oxidative pyrolysis of 1-methylnaphthalene in the presence of Fe(III)(2)O-3 nanoparticles. *Combust. Flame* **2013,** *160* (12), 2996-3003.
8. Mohamad Barekati-Goudarzi; Dorin Boldor; Marculesc, C.; Lavrent Khachatryan, The Peculiarities of Pyrolysis of Hydrolytic Lignin in Dispersed Gas Phase and in Solid State. *Energy Fuels* **2017,** *31 (11)*, 12156-12167.
9. Thibodeaux, C. A., The electronic structure of environmentally persistent free radicals formed on metal oxide surfaces. *Ph.D. Dissertation* **2015,** *McNeese State University*.
10. D'Arienzo, M.; Gamba, L.; Morazzoni, F.; Cosentino, U.; Greco, C.; Lasagni, M.; Pitea, D.; Moro, G.; Cepek, C.; Butera, V.; Sicilia, E.; Russo, N.; Munoz-Garcia, A. B.; Pavone, M., Experimental and Theoretical Investigation on the Catalytic Generation of Environmentally Persistent Free Radicals from Benzene. *J. Phys. Chem. C* **2017,** *121* (17), 9381-9393.
11. Vejerano, E. P.; Rao, G. Y.; Khachatryan, L.; Cormier, S. A.; Lomnicki, S., Environmentally Persistent Free Radicals: Insights on a New Class of Pollutants. *Environ. Sci. Technol.* **2018,** *52* (5), 2468-2481.
12. Valeria B. Arce; Janina A. Rosso; Fernando J. V. E. Oliveira; Claudio Airoldi; Delia B. Soria; Mo´nica C. Gonzalez; Patricia E. Allegretti, a.; Ma´rtire, D. O., Generation of Chemisorbed Benzyl Radicals on Silica Nanoparticles. *Photochem. Photobiol.* **2010,** *86*, 1208-1214.
13. (a)Borges dos Santos, R. U.; Martinho Simoes, J. A.; *J. Phys. Chem. Ref.Data* 1998, 707-741; ; (b) Fattahi, A.; Kass, S. R.; Liebman, J. F.; Matos, M. A. R.; Miranda, M. S.; Morais, V. M. S.; *J. Amer. Chem. Soc., 127,* 6116.
14. Stoll, S.; Ozarowski, A.; Britt, R. D.; Angerhofer, A., Atomic hydrogen as high-precision field standard for high-field EPR. *J. Magn. Reson.* **2010,** *207* (1), 158-163.

15. Khachatryan, L.; Adounkpe, J.; Maskos, M., and ; Dellinger, B., Formation of Cyclopentadienyl Radicals from the Gas-Phase Pyrolysis of Hydroquinone, Catechol, and Phenol. *Environ. Sci. Technol.* **2006,** *40*, 5071-5076.
16. Asatryan, R.; Bennadji, H.; Bozzelli, J. W.; Ruckenstein, E.; Khachatryan, L., Molecular Products and Fundamentally Based Reaction Pathways in the Gas-Phase Pyrolysis of the Lignin Model Compound p-Coumaryl Alcohol. *J. Phys. Chem. A* **2017,** *121* (18), 3352-3371.
17. Barekati-Goudarzi, M.; Boldor, D.; Khachatryan, L.; Lynn, B.; Kalinoski, R.; Shi, J., Heterogeneous and Homogeneous Components in Gas-Phase Pyrolysis of Hydrolytic Lignin. *ACS Sustainable Chem. Eng.* **2020,** *8* (34), 12891-12901.
18. Smith, E. A.; Lee, Y. J., Petroleomic Analysis of Bio-oils from the Fast Pyrolysis of Biomass: Laser Desorption Ionization-Linear Ion Trap-Orbitrap Mass Spectrometry Approach. *Energy Fuels* **2010,** *24*, 5190-5198.
19. Sanchez-Gonzalez, A.; Martin-Martinez, F. J.; Dobado, J. A., The role of weak interactions in lignin polymerization. *J. Mol. Model.* **2017,** *23* (3).
20. Benson, S. W. Thermochemical Kinetics; Methods for the Estimation of Thermochemical Data and Rate Parameters. 2nd ed. John Wiley & Sons, Inc., New York **1976**.
21. Asatryan, R.; Davtyan, A.; Khachatryan, L.; Dellinger, B.; Molecular modeling studies of the reactions of phenoxyl radical dimers: pathways to polychlorinated dibenzofurans, *J. Phys. Chem. A* **2005,** *109*, 49, 11198–11205.
22. Asatryan, R.; Hudzik, J.M.; Bozzelli, J.W.; Khachatryan, L.; Ruckenstein, E. OH initiated reactions of p-coumaryl alcohol relevant to the lignin pyrolysis. Part I. potential energy surface analysis, *J. Phys. Chem. A* **2019**, *123*, 2570–2585.
